# Response to Commentary on “Force-field functor theory: classical force-fields which reproduce equilibrium quantum distributions”

**DOI:** 10.3389/fchem.2013.00033

**Published:** 2013-12-24

**Authors:** Ryan Babbush, John A. Parkhill, Alán Aspuru-Guzik

**Affiliations:** ^1^Department of Chemistry, Harvard UniversityCambridge, MA, USA; ^2^Parkhill Group, Department of Chemistry, The University of Notre DameSouth Bend, IN, USA

**Keywords:** path integral molecular dynamics, density functional Theory, effective potentials, quantum propagation, path integral monte carlo

Dr. Ruggero Vaia has made several claims (Vaia, 2013) about our recent submission entitled *Force-field functor theory: classical force-fields which reproduce equilibrium quantum distributions* (Babbush et al., 2013) which we would like to address. Dr. Vaia points out that the idea of an effective potential which reproduces quantum distributions is a well-known observation. We agree and point out that our paper contains many citations to the relevant literature. The contribution we make is to describe a new perspective on how the effective potential might be obtained in a spirit similar to Density Functional Theory (DFT). To make progress on a DFT-like theory, a proof of the existence and uniqueness of force-field functors is necessary. The main goal of our manuscript is to develop such proofs and to demonstrate and test the idea of functor on various test problems.

After establishing uniqueness and existence proofs, we introduce an intentionally simple example of a functor in order to illustrate the utility of our broader approach. In particular, we use the Jensen–Peierls inequality to construct a variational approximation in which the mapping is linear. This procedure closely parallels the effective potential of Feynman and Hibbs (1965) in which the same approximation is used to model the effective potential as a *linear* convolution of the physical potential with a Gaussian of variance $\sigma^{2}=\frac{\beta \hbar^{2}}{12}$ m. Dr. Vaia correctly shows that *for a one-dimensional system*, our analytical approximation reduces to a Gaussian smearing of the potential with exactly twice the width of the Feynman–Hibbs approximation. This is because the Feynman–Hibbs potential is a centroid effective potential which does not attempt to reproduce the correct particle distribution. While we make reference to this in our paper (we wrote “our [linear mapping] can be imagined as a Gaussian smearing of *V*(*q*)”), we do not discuss the explicit form of the mapping in one-dimension as this obscures the more general point we are trying to make.

Linearity is not an essential feature of our approach; rather, it is an approximation which underlies a particular functor that we construct as a pedagogical example of how the functor approach could be used. In fact, the procedure we demonstrate to invert the linear mapping is significantly more accurate than the explicit Gaussian convolution which Dr. Vaia discusses. We used the bijective nature of the mapping (implied by our uniqueness proof) to empirically fit the optimal linear functor. This was accomplished by computing the exact effective potentials for 1000 random physical potentials and then using a least squares procedure with cross-validation to determine optimal matrix elements which map basis vectors of the physical potential to basis vectors of the effective potential. This cross-validation approach allowed us to test the accuracy of our method. We show that our functor works surprising well (especially considering its simplicity) and demonstrate its impressive performance on independent test problems including the simulation of liquid hydrogen at temperatures as low as 14 K. Though the error in this approximation grows as temperature decreases, the same is true of all path integral approaches since the number of required integration time slices grows without bound as temperature approaches zero. Degrading in quality at *T* = 0 does not render path integral molecular dynamics, or our technique useless.

Dr. Vaia goes on to compare the linear approximation to the Feynman–Kleinert and Giachetti–Tognetti effective potentials and mentions that those approximations are more robust. While this is true, *we are not trying to demonstrate the superiority of a particular explicit form for the functor under any particular approximation and we are certainly not interested in comparing the performance of this simple example to other paradigm methods in one dimension*. The linear functor is for our theory analogous to what Local Density Approximation (LDA) is for DFT: a simplistic but illustrative example of the type of theory which could emerge from our uniqueness theorem. We have provided ample evidence for a new approach to obtaining effective potentials, in a similar spirit to DFT.
